# Extracting Vehicle Trajectories from Partially Overlapping Roadside Radar

**DOI:** 10.3390/s24144640

**Published:** 2024-07-17

**Authors:** Maxwell Schrader, Alexander Hainen, Joshua Bittle

**Affiliations:** Department of Mechanical Engineering, The University of Alabama, Tuscaloosa, AL 35487, USA; mcschrader@crimson.ua.edu (M.S.); ahainen@eng.ua.edu (A.H.)

**Keywords:** data fusion, trajectory calibration, partially-overlapping fusion, car-following model, driver behavior

## Abstract

This work presents a methodology for extracting vehicle trajectories from six partially-overlapping roadside radars through a signalized corridor. The methodology incorporates radar calibration, transformation to the Frenet space, Kalman filtering, short-term prediction, lane-classification, trajectory association, and a covariance intersection-based approach to track fusion. The resulting dataset contains 79,000 fused radar trajectories over a 26-h period, capturing diverse driving scenarios including signalized intersections, merging behavior, and a wide range of speeds. Compared to popular trajectory datasets such as NGSIM and highD, this dataset offers extended temporal coverage, a large number of vehicles, and varied driving conditions. The filtered leader–follower pairs from the dataset provide a substantial number of trajectories suitable for car-following model calibration. The framework and dataset presented in this work has the potential to be leveraged broadly in the study of advanced traffic management systems, autonomous vehicle decision-making, and traffic research.

## 1. Introduction

Intelligent Transportation Systems (ITS) have driven a surge in the deployment of roadside millimeter-wave radars (MMW radar), a technology that has become increasingly ubiquitous in modern traffic management systems. The spatially and temporally resolved stream of information that radars provide is useful for many classical aggregated traffic applications, including queue length estimation, advanced detection, and measuring link-average speed. The measurements can be passed into traffic management systems which aim to improve traffic flow or reduce the traffic system’s emissions. When the radar is combined with vehicle-to-everything communication (V2X)-based applications, the radars can augment an autonomous vehicle’s sensor suite field-of-view (FoV), aiding in decision making [1]. From a traffic research perspective, the recorded vehicle trajectories can be used for microscopic car-following model calibration, fuel consumption and emissions estimation, lane-change modeling, and traffic flow studies [2,3].

Radars are not the only sensing technology deployed at the roadside; in fact, they fit into the broader classification of roadside perception units, along with cameras, LiDAR, and other roadside-units (RSUs). Each sensor type has its own benefits and shortcomings, which can be mitigated through sensor fusion. Commonly fused sensors in literature are camera and radar [4] and camera and LiDAR, as radar and LiDAR performing well in all lighting conditions, whereas cameras suffer in bad lighting but are better suited for identifying vehicle size and type [5].

The use of multiple sensors necessitates data fusion. In practice, there are different forms of fusion, including raw/low-level fusion, feature-based fusion, and track-level fusion [6]. The literature in the transportation space typically focuses on low-level fusion with the same FoV, whereas many automotive applications have pivoted to track-level fusion, which allows integrators to abstract the implementations of the sensor-specific pipelines and focus on the task of associating the tracks from each sensor [7].

In comparison to the prior literature on single-FoV problems, this work focuses the task of fusing trajectories from six (6) radars in a 1.5 km corridor. The radar FoVs theoretically overlap; however, because tracking accuracy is not perfect, the radars rarely track objects completely through their FoV. Instead, trajectories must be extended with a short-term (4 s) prediction. The prediction, filtering, and subsequent matching all occur in the Frenet coordinated system. This allows for implicit injection of road information into the system, reducing lateral noise in measurements that can impact matching results. While the same methods could be applied to a real-time system, the aim of this work is to extract vehicle trajectories for traffic flow studies and car-following model calibration; thus, it is written in a postprocessing context.

The remainder of the paper first presents a literature review, then a novel methodology for extracting complete vehicle trajectories (i.e., the complete path through the 1.5 km corridor) by fusing tracked objects from six radars, with the assumption that radar uncertainty is obfuscated from the end user. The methodology encompasses extraction of data, radar calibration, transformation to the Frenet space, Interacting Multiple Model (IMM) filtering, lane-classification, short-term prediction, trajectory association, and fusion. The result is a feature-rich trajectory dataset including lane-changes, merging, car-following interactions, and the impact of traffic signals. The methodology is validated using a probe vehicle equipped with a differential GPS. Finally, a summary of the dataset is provided, along with a quantitative comparison to several common trajectory datasets.

## 2. Background

### 2.1. Trajectory Datasets

The field of transportation engineering has seen a surge in publications related to roadside-sensor fusion, also known as infrastructure-based fusion, as researchers grapple with the outputs of ITS [8]. The fusion of data from these sensors is crucial for several reasons, including enhancing system reliability, merging heterogeneous data sources, improving system prediction accuracy, and extending the FoV of individual sensors [8,9]. Still, a survey of literature indicates that the application of roadside-sensor fusion to a corridor with multiple partially overlapping sensors is a novel problem in the literature. (searches for “radar” AND “fusion” AND “roadside”) There are several works focused on the fusion of roadside radars and roadside cameras; however, these generally have overlapping FoVs and are deployed at the same origin [5,10,11]. Efforts based on single radars have tackled the problems of calibration [12] and missing track imputation [13] through car-following modeling approaches.

As the fused trajectories in this work represent vehicle trajectories on a 1.5 km path, they can be used for many of the same tasks as popular trajectory datasets such as NGSIM [14] and highD dataset [15], both of which are recorded from cameras (highD from a drone) and postprocessed to extract trajectories. The corridor of study has several advantages over those datasets, including complex traffic interactions at signalized intersections, merging behavior from side-street entrances, and data coverage during periods of free-flow traffic as well as peak-hour congestion, topics that a review of trajectory datasets indicates has been missing [16]. Prior works have postulated that driver behavior evolves over time or changes depending on geography and locality [17], making the extraction of localized trajectories a useful task for the authors. Further, the methods developed here can be used by others to evaluate locality effects for comparison and analysis of regional variability.

### 2.2. Data Fusion

To construct a trajectory dataset that extends past the FoV of any one sensor, data association and fusion must be performed. In addition to trajectory dataset creation, association and fusion is a critical component in realizing the potential of ITS, as it improves object tracking by reducing uncertainty and expanding the FoV of a single sensor. For instance, roadside radars have been shown to display high positional uncertainty when vehicles are near the sensor, as they typically use a central FoV aiming for longer-distance applications. By fusing the radar data with positional information from a camera feed, the fused positional information can achieve higher accuracy than either system could achieve independently while also extending their respective ranges [4].

The field of data association has been extensively studied, particularly in the aerospace and automotive industries [18]. These sectors have pioneered the application of data association techniques, and broadly break down the tasks of data fusion into two steps: association, which maps a measurement (or track) from one sensor to another sensor’s measurement, and fusion, which joins mapped measurements (or tracks) from each of the sensors into one jointly estimated object state. As with most modern applications, fusing roadside radars occurs in a multi-target environment that includes clutter, making the goal of this work a multi-target tracking system (MTT). The fundamental problem in MTT is identifying which measurements belong to which track, if any (i.e., distinguishing clutter), which incurs high computation cost [19].

Classically, data association and fusion happen at the measurement level; this is often called raw or low-level fusion. It is the optimal association strategy from an information-theoretical perspective, as no data reduction takes place and incidental process noise correlations are not introduced [20]. However, the implementation requires access to raw sensor measurements and can incur high computation and bandwidth costs, in addition to being sensitive to sensor uncertainty, calibration and timing [6,7]. There are several well known methods for multi-sensor fusion and association in an MTT system, including global nearest neighbor (GNN), joint probabilistic data association (JPDA), and Markov chain Monte Carlo data association (MCMCDA) [21].

Track-to-track fusion (T2TF) is based on the premise that each sensor executes its own low-level data association and tracking algorithm. The sensor’s output is a list of tracks, each comprising estimated state variables, the associated covariance, and an object identifier for chaining measurements over time. These tracks may originate from sensors with identical or different state spaces, referred to as homogeneous and heterogeneous T2TF, respectively. A centralized node can associate and fuse these tracks at either synchronous or asynchronous intervals. T2TF offers advantages over low-level fusion such as increased modularity and reduced computational and networking load, due to the decrease in information transmitted from the sensor to the association node [20]. However, as in low-level data fusion, the integration of tracks from multiple sensors in a multi-target tracking (MTT) environment necessitates association and fusion steps. Various track-to-track association (T2TA) strategies have been proposed, including a track-to-track Mahalanobis distance metric utilizing track history [22] and a permutation matrix-based approach minimizing the spatial, temporal, and mismatch costs [7]. In automotive applications, T2TF typically employs a GNN approach [23].

Building upon the Mahalanobis approach, the authors of [22] computed the average modified Mahalanobis distance over the history of two tracks up to the present moment. This is expressed as

(1) $$d_{assoc}^{2}={\left(z_{i}-z_{j}\right)}^{T}S_{i,j}^{-1}\left(z_{i}-z_{j}\right)+\ln\left(|S_{i,j}|\right)$$

where $z_{i}$ and $z_{j}$ represent measurements from two distinct tracks and $S_{i,j}$ is the covariance matrix of the measurement noise. The inclusion of the normalization factor $\ln\left(|S_{i,j}|\right)$ serves to mitigate the likelihood of large covariance matrices resulting in small Mahalanobis distances, thereby preventing incorrect associations. This was formalized in the association log-likelihood distance, proposed by [24] and implemented in a T2T scenarios in [22,25].

After association, the tracks can be fused using various algorithms. One of the most prevalent methods, particularly when the correlation between tracks is unknown or cannot be accurately determined, is covariance intersection (CI) [26]. CI approximates the correlation between tracks by geometrically fusing their state estimates and covariance [26,27]. A special case of CI is the two-sensor case, where the updated mean $\hat{x}\left(t|t\right)$ and covariance $P\left(t|t\right)$ at time *t* for sensors *i* and *j* are estimated by

(2) $$P{\left(t|t\right)}^{-1}=\omega P_{i}{\left(t|t\right)}^{-1}+\left(1-\omega \right)P_{j}{\left(t|t\right)}^{-1},$$

(3) $$\hat{x}\left(t|t\right)=P\left(t|t\right)\left(\omega P_{i}{\left(t|t\right)}^{-1}x_{i}+\left(1-\omega \right)P_{j}{\left(t|t\right)}^{-1}x_{j}\right),$$

where $P_{i}\left(t|t\right)$ and $x_{i}$ represent the covariance and state estimate of sensor *i* at time *t* and $P_{j}\left(t|t\right)$ and $x_{j}$ represent the same for sensor *j*. The notation t|t indicates that the covariance and state estimates are the result of a Kalman filter *update* step, also know as the a posteriori estimate. The weighting factor ω is found by minimizing the determinant or trace of Equation (2):

(4) $$\omega =\min_{\omega }\left(\det\left(P(t|t)\right)\right)$$

where $\omega \in \left[0,1\right]$ [26]. When more than two sensors are present (n>2), the equation can be solved recursively, resulting in an n−1-dimensional optimization problem [28]. Equation (4) is a nonlinear function, making the computation of ω computationally intensive. Because the optimization of ω requires the minimization of a nonlinear function, several alternatives have been proposed, including fast covariance intersection [29] and improved fast covariance intersection [30], which simplify the calculation of ω to a ratio of covariance matrix traces and determinants, respectively.

The original presentation of CI was in a distributed T2TF application, meaning that each sensor fuses the other sensors’ information. As applied to this work, a global track for each vehicle must be maintained as the vehicle moves through the FoVs of different radars. Following [27], Equations (2) and (3) are modified to incorporate information from the global track:

(5) $$P{\left(t|t\right)}^{-1}=\omega P{\left(t|t-1\right)}^{-1}+\left(1-\omega \right)P_{i}{\left(t|t\right)}^{-1}$$

(6) $$\hat{x}\left(t|t\right)=P\left(t|t\right)\left(\omega P{\left(t|t-1\right)}^{-1}\hat{x}\left(t|t-1\right)+\left(1-\omega \right)P_{i}{\left(t|t\right)}^{-1}x_{i}\right)$$

where $P\left(t|t-1\right)$ and $\hat{x}\left(t|t-1\right)$ represent the globally fused covariance and state, respectively, from time t−1 predicted to time *t* using the Kalman prediction equations. As a vehicle traverses the network, the global track is initially the same as that of the first radar to pick up the vehicle’s track. As more information from other radars becomes available, it is fused into the global track using CI, which results in some deviation from the track of a single radar.

### 2.3. Frenet Coordinate System

The association and fusion referenced above is typically applied in the Cartesian space, which can be relative to the sensor or projected to a global plane. In the case of vehicle tracking on roadways, however, vehicles are generally not free to move in any direction, as assumed in Cartesian-based tracking; instead, drivers follow the roadway and stay inside the bounds of the lane markings. Further, it is often convenient for downstream applications to categorize vehicles by lane or to estimate the travel time to the destination, which is generally not calculated by knowing the Euclidean distance from the destination but rather requires knowledge of the *path*. To incorporate this roadway knowledge into the coordinate system, several works have suggested implementing a *curvilinear* or so-called Frenet frame [31,32,33].

**Figure 1:**
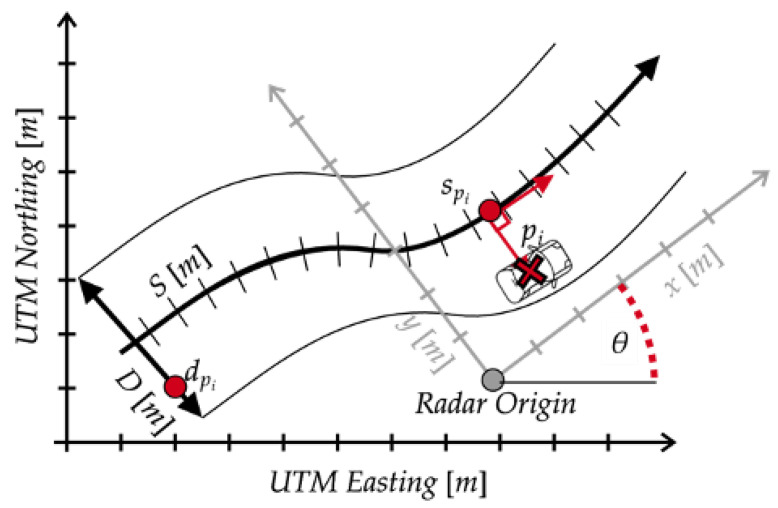
Overlay showing three coordinate systems: UTM, Radar Relative, and Frenet.

Figure 1 displays the Frenet frame, where a Cartesian position in (x,y) relative to the radar is mapped to a distance *s* along the lane’s center line and the orthogonal distance from the center line *d*. The orthogonality of the two new dimensions *s* and *d* makes it straightforward to track motion-related problems constrained on the curvilinear coordinates [31]. Owing to its simplicity, the Frenet frame is frequently used in the path prediction and planning literature for autonomous vehicles and robots [34]. From a traffic research perspective, the Frenet frame simplifies identification of lane changes, as lanes on multi-lane roads can be identified using only the *D* dimension. Further, the Frenet frame minimizes lateral drift when performing Kalman filter-based short-term predictions, a feature on which this work relies.

While the use of the Frenet frame offers several advantages in motion modeling, it also presents certain limitations. Lanes center lines are typically modeled using a B-spline model. Mapping Cartesian coordinates to the nearest perpendicular position $c_{p}$ on the *S* axis in a continuous space requires solving a quadratic minimization problem; thus, in order to have a Frenet frame at all, high-quality mapping data must be available, as even a small relative error (0.45 m) in mapping the center line has been shown to lead to tracking errors, especially when using the Frenet coordinates to classify lane change behavior [31]. Further, there can be ambiguity when multiple “lanes” intersect, such as at a zipper junction.

Despite these acknowledged limitations, the use of Frenet coordinates in roadside applications mitigates some of these concerns. First, practitioners only need a high-quality map of the sensor FoV, whereas using the Frenet frame in an autonomous vehicle application necessitates a high-quality map of the entire trip. Second, because the FoV is constrained, and consequently the *S* axis as well, the quadratic minimization problem in mapping (x,y)→(S,D) can be reasonably discretized and solved with the Euclidean distance using a binary search tree.

## 3. Methods

### 3.1. Network and Hardware

The six (6) radars in this work are the iSYS-5220 model from InnoSenT GmbH (https://www.innosent.de/, accessed on 2 July 2024), which is part of Econolite’s EVO system (https://www.econolite.com/solutions/sensors/evo-radar/, accessed on 2 July 2024). This radar performs onboard data association and fusion to output vehicle tracks via an API. The specifics of InnoSenT’s internal algorithms are not publicly available; thus, the system is treated as a black box for the purposes of this work. Data stream recording for all six radars was in parallel at an interval of 75 ms, and the streams were recorded synchronously on a central computer. The recorded data included vehicle position, velocity, heading, and length.

In postprocessing, the radar data were downsampled to a period of 100 ms using the interval mean. Included in the data of each radar track is a flag for whether or not the reported object values come from the radar’s internal prediction. While the specifics of the radar prediction are not described in the manufacturer’s documentation, it is apparent from analysis of the data that a constant velocity extrapolation (in the radar’s Cartesian coordinate system) is used for 5 s whenever an object is lost by the radar. As the internal model used to make these predictions is unknown, the predictions at the end of the tracks were removed.

**Figure 2:**
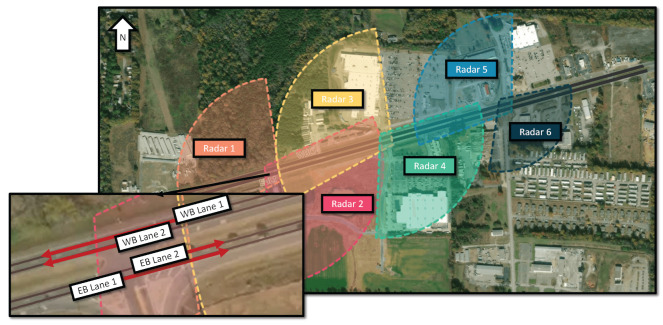
The network of interest, shown with the FoV of all six (6) radars. The colored region is drawn to the 95th percentile of range. The exploded intersection shows the lane center lines used as the Frenet frames. The centroid of the network is located at (33.235, −87.614).

The radars were deployed at three consecutive signalized intersections in Tuscaloosa, Alabama, USA. The deployment is illustrated in Figure 2, which shows a region centered at (33.235°, −87.614°). The shaded regions depicted in the diagram serve as a visual representation of the FoV for each of the six radars under consideration. These regions were constructed with a radius corresponding to the 95th percentile of the observed vehicles’ distance from each radar’s origin (327 m on average). The average track was maintained for 146 m, which does not extend through the radar range due to tracking issues and the geometry of the roadway. Though the main east–west roadway is divided in Figure 2, all radars captured vehicles in both directions, as the median does not impact the FoV. The radars were rotated to the UTM coordinates in Figure 2 based on the calibrated angles of rotation following the process described in Section 3.3. The location of each radar head was surveyed within 1 cm accuracy using a differential GPS system.

The majority of traffic within the network moves from east to west during the morning hours, then reverses its course from west to east in the afternoon, as this is a main artery linking residential areas to the west with the city of Tuscaloosa to the east. The east–west road is a four-lane divided state highway, which is illustrated in the exploded view of Figure 2. These four lanes formed the primary focus of the study, as the objective was to extract comprehensive trajectories of vehicles as they traverse the network. Therefore, in the data fusion process we have chosen to concentrate solely on the lanes drawn in black.

The center lines were also captured using the differential GPS system installed on a probe vehicle. This vehicle was driven down the center of each of the four lanes to the best of the driver’s ability, capturing coordinates with an accuracy of ±0.02 m (assuming a perfect drive). The lanes under investigation were straight, exhibiting negligible curvature throughout the corridor. Despite the straightforward nature of these lanes, the methodology was designed to be applicable to a variety of scenarios. The trajectories were simplified using Chaikin’s corner-cutting algorithm. The simplified geometry was then interpolated to the desired resolution.

As depicted in Figure 2, all four lanes were incorporated into the calibration process; however, only the two rightmost lanes, one in each direction, were utilized as the *s* axes in the Frenet coordinate system for matching. This approach was chosen in order to maintain continuity in the *s* space between lanes. By using only one Frenet axis per road, it was possible to model lane change maneuvers using the Kalman filtering approach.

### 3.2. Coordinate Conversion

Fusing multiple sensors together involves projection to a common coordinate system. In this work, there are two projections, first from each radars’ local coordinate system to Universal Transverse Mercator (UTM), and second from UTM to the Frenet coordinate system. The first step requires mapping the radar origin to UTM coordinates as well as the angle of rotation θ from the radar’s *x*-axis to the UTM Easting axis, which is displayed in Figure 1. The calibration process for θ is described below in Section 3.3. Given that θ is known for each radar, the coordinate conversion for point $p^{i}$ can be written as

(7) $$f_{\mathrm{radar}\;\to \mathrm{UTM}}=\left[\begin{array}{cc} \cos(\theta_{j}) & -\sin(\theta_{j})\\ \sin(\theta_{j}) & \cos(\theta_{j}) \end{array}\right]\left[\begin{array}{c} p_{x}^{i}\\ p_{y}^{i} \end{array}\right]+\left[\begin{array}{c} X_{j}\\ Y_{j} \end{array}\right],$$

where $\left(X_{j},Y_{j}\right)$ is the UTM position of radar *j* and $\theta_{j}$ is the angle of rotation. With the radar data in UTM coordinates, the state vector of an object *i* at an arbitrary time *t* can be represented as

(8) $${\left[\begin{array}{c} x\;y\;v\;\epsilon \end{array}\right]}_{i_{t}},$$

where ϵ is the vehicle’s heading angle rotated to align with the UTM Easting axis. The relationship between the radar coordinate system and UTM is visually represented in Figure 1.

Upon transformation of the radar data into UTM coordinates, it is subsequently mapped onto Frenet coordinates. To enhance processing efficiency, the Frenet axes, represented by the lane center lines, undergo a discretization process, resulting in a UTM coordinate every 0.01 m along the path. A K-D tree [35] incorporating the points from the desired lanes is then constructed. This tree is queried based on the Euclidean distance to find the nearest lane and the closest point on the Frenet axis for each respective position.

After the closest point on the center line has been found, the measured Frenet state becomes

(9) $${\left[\begin{array}{c} s\;\dot{s}\;d\;\dot{d} \end{array}\right]}_{j_{t}},$$

where $\dot{s}$ is calculated as the component of the velocity vector in the direction of the Frenet axis, *s* is the path distance from the origin on the Frenet lane, *d* is the perpendicular distance from the lane centerline, and $\dot{d}$ is the component of the velocity perpendicular to the Frenet axis. The *d* distance (and $\dot{d}$) is signed, using the minor angle from the center line to the measurement location.

The transformation of coordinates is a reversible process, which is achieved by mapping the *s* measurement to the approximate ${\left(x,y\right)}_{\mathrm{lane}}$ point on the center line. Subsequently, the bearing angle of the road at that specific location is utilized to adjust the ${\left(x,y\right)}_{\mathrm{lane}}$ position employing the *d* distance and its respective *x* and *y* components. Due to the inherent discretization, the conversion from the UTM position to the Frenet frame and vice versa is not entirely lossless; however, with lane discretization at every 0.01 m, the round trip conversion yields a mean positional error of 0.0025 m. This magnitude of error is considerably lower than the uncertainty attributed to radar and calibration processes.

### 3.3. Rotation Calibration

The conversion of radar to UTM coordinates, as outlined in Section 3.2, requires the radar origin in UTM coordinates along with the rotation angle, $\theta_{i}$, the elevation angle, and the installation height. The elevation angle and installation height are assumed to be known, leaving the rotation angle and the radar origin (*x*, *y*) as calibration parameters. This calibration process is designed as an optimization problem, where the ideal θ, *x*, and *y* are found by minimizing the mean Euclidean distance between the vehicle positions base on the radar and the lane center lines. This method is based on the presumption that, given a large enough calibration dataset, vehicles will on average follow the center lines.

For optimization, the center lines of the lanes in Figure 2 were used, along with the center lines of the side streets. A sample of data from the radar was matched to the center lines using the discretization approach outlined in Section 3.2, and the average *d* error was found. In addition to the average *d* distance, the percentage of matched points lying within a threshold distance of the center lines was calculated. A term was added to the calibration objective function to maximize the percentage of points inside of the threshold, helping to prevent the rotation calibration from finding suboptimal solutions. Calibration was applied to the six radars independently using the Nelder–Mead optimization algorithm, as shown in Figure 2. The data were filtered to only include measurements within the radars’ range of 250 m.

### 3.4. Interacting Multiple Model Filtering

The radar does not make the covariance matrix accessible in the API, nor is measurement uncertainty directly available. As alluded to in Section 2.2, using CI necessitates the existence of a covariance matrix, P. To overcome this, an Interacting Multiple Model (IMM) filter [36] was utilized to create the uncertainty approximations and to fill gaps where radar data were missing. The filter was implemented after the conversion into the Frenet coordinated system in order to exploit the resulting geometric advantages. The IMM filter enhances the conventional Kalman filter by running multiple Kalman filters in parallel, each considering a different prediction model hypothesis. The equations for general predictions for a single linear Kalman filter are as follows:

(10) $$\begin{array}{cc} x_{t|t-1} & =F_{t}x_{t-1|t-1} \end{array}$$

(11) $$\begin{array}{cc} P_{t|t-1} & =F_{t}P_{t-1|t-1}F_{t}^{T}+Q_{t} \end{array}$$

where $x_{t|t-1}$ represents the predicted state estimate, $P_{t|t-1}$ is the predicted covariance matrix, $F_{t}$ is the state transition model, and $Q_{t}$ is the process noise covariance. After prediction, the Kalman filter performs an update step in which the residual between the predicted state $x_{t|t-1}$ and a measurement $z_{t}$ is calculated, followed by the Kalman gain, and ultimately the a posteriori state $x_{t|t}$ and covariance matrix $P_{t|t}$.

The linear Kalman filter, while effective in many scenarios, encounters limitations when tracking maneuvering targets. This is primarily due to its inherent assumption of a linear Gaussian model, which may not accurately represent the dynamics of maneuvering targets. The IMM filter adopts a probabilistic approach to address this limitation, amalgamating the outputs of multiple internal Kalman filters to provide a more robust solution for tracking maneuvering targets. The operation of the IMM filter is divided into three primary steps: interaction, probability update, and mixing. The interaction step serves as the initial phase, in which the model probability, $\mu_{t-1}$, and the transition probability matrix, $\pi_{t-1}$ are utilized to compute the mixed initial conditions for each model. This step prepares the system for potential changes in the target’s motion model by generating a set of mixed initial states and covariances for each model.

Following the interaction step, each filter independently predicts the target’s state and updates the estimate based on the measurement. This process allows the IMM filter to maintain multiple hypotheses about the target’s motion, thereby enhancing its ability to track maneuvering targets. Subsequently, the model probabilities μ are updated using the likelihood of each Kalman filter. This probability update step ensures that the most likely model is given more weight in the final state estimate. In the final step, the IMM filter combines the state estimates from all of the filters using a Gaussian mixture equation. This mixing step generates a single optimal estimate that is a comprehensive representation of all the information available to the IMM filter.

A unique feature of the IMM filter is its ability to not only predict the state of the tracked object but also maintain $\mu_{t}$, which essentially represents the likelihood of the tracked object’s motion being defined by each internal motion model. This feature enables the IMM filter to categorize vehicle behavior, including lane changes and acceleration versus constant velocity driving [32]. Thus, the IMM filter enhances the overall performance of the radar system by providing more accurate and robust tracking of maneuvering targets.

In this study, the Interacting Multiple Model (IMM) filter incorporates three motion models: *constant velocity lane-keeping* (CVLK), *constant acceleration lane-keeping* (CALK), and *constant acceleration lane-changing* (CALC). These models are based on the work of [31]. The CALC model is expressed as follows:

(12) $${\left[\begin{array}{c} s\\ \dot{s}\\ \ddot{s}\\ d\\ \dot{d}\\ \ddot{d} \end{array}\right]}_{t}=\left[\begin{array}{cccccc} 1 & \Delta t & \frac{\Delta t^{2}}{2} & 0 & 0 & 0\\ 0 & 1 & \Delta t & 0 & 0 & 0\\ 0 & 0 & 1 & 0 & 0 & 0\\ 0 & 0 & 0 & 1 & \Delta t & \frac{\Delta t^{2}}{2}\\ 0 & 0 & 0 & 0 & 1 & \Delta t\\ 0 & 0 & 0 & 0 & 0 & 1 \end{array}\right]{\left[\begin{array}{c} s\\ \dot{s}\\ \ddot{s}\\ d\\ \dot{d}\\ \ddot{d} \end{array}\right]}_{t-1}+Q\left(w_{a_{s}},w_{a_{d}}\right)$$

where $w_{a_{s}}$ and $w_{a_{d}}$ are zero-mean Gaussian distributions $N\left(0,\sigma_{a_{s}}^{2}\right)$, $N\left(0,\sigma_{a_{d}}^{2}\right)$ and $Q(w_{a_{s}},w_{a_{d}})$ represents the discrete constant white noise model. The process noise in the longitudinal direction encapsulates both the acceleration and deceleration capabilities of the vehicle. Similarly, the standard deviation of the noise in the lateral direction $\left(\sigma_{a_{d}}\right)$ captures the lateral acceleration. For a comprehensive understanding of the full state space equations of each of the remaining models, the reader is referred to [31].

All motion models in the IMM are linear, and are applied to the radar data using the standard Kalman filter prediction and update equations. With a discretization step size of 0.01 m, the uncertainty $\sigma_{s}$ in the *s* axis is $\frac{ds}{2}$, or 0.005 m. As the radar system’s uncertainty is unknown, the measurement uncertainty covariance matrix of the internal Kalman filters assume typical measurement uncertainty for similar systems (Comparable smartmicro datasheet, https://www.smartmicro.com/fileadmin/media/Downloads/Traffic_Radar/Sensor_Data_Sheets__24_GHz_/TRUGRD_UMRR-12_Type_48_Traffic_Management_Datasheet.pdf, accessed on 2 July 2024) The radars in the system do not consistently track vehicles to the edge of their FoV, resulting in situations where a vehicle track from one radar drops before another radar begins tracking the vehicle. To address this issue, a short-term trajectory prediction for each vehicle is generated using the Interacting Multiple Model (IMM) filter for a duration of 4 s. In this prediction, the model probability of lane changes ($\mu_{\mathrm{CALC}}$) and acceleration ($\mu_{\mathrm{CALK}}$) are set to zero in order to minimize lateral and longitudinal prediction errors, ensuring a more accurate trajectory estimation. As constant velocity assumed, short-term predictions are most accurate when vehicle behavior is best captured by the CVLK model. The prediction duration of 4 s was determined through a comprehensive analysis of the dataset, striking a balance between maximizing the number of correctly associated trajectories and minimizing the occurrence of incorrect matches arising from the CVLK assumption.

**Figure 3:**
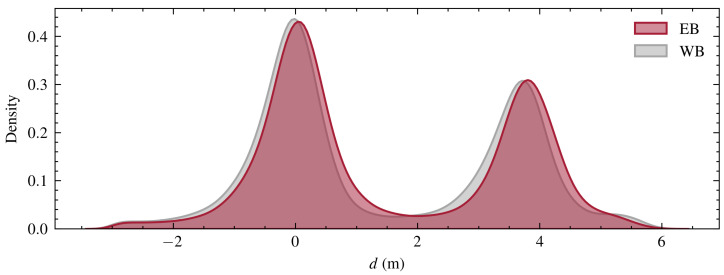
Distribution of *d* measurements from the rightmost lane in both the EB and WB directions, where traffic flow can be considered as coming towards the reader.

Although the IMM filter and prediction mechanism are employed within the context of a single-lane-per-direction map, the subsequent association strategy must determine whether a vehicle occupies Lane 1 or Lane 2 in each direction, as illustrated in Figure 2. To achieve this, the *d* dimension of the filtered trajectory is mapped to the nearest lane. Figure 3 presents the distribution of *d* for both eastbound (EB) and westbound (WB) traffic, providing insights into the lane occupancy patterns within the network. This lane-level information is crucial for accurately associating vehicle trajectories across different radar FoVs and constructing comprehensive vehicle paths through the network.

The bimodal distributions in Figure 3 represent the two lanes in each direction of travel. The figure highlights the preference of drivers to use the rightmost lane, as well as a slight discrepancy in means of bimodal distribution, which is indicative of less than perfect lane center line identification.

### 3.5. Association

Traditional data association is typically approached as a combinatorial matching problem, often simplified through the use of gating techniques [37]. However, this paper proposes a simplified optimistic approach to the problem, leveraging the geometry of the roadway and making several key assumptions to streamline the process. This approach should be directly applicable by others with similar roadside detection hardware.

The first assumption is that for any given follower vehicle, denoted as *f*, there can only be one leader vehicle, denoted as *l*, at any given time *t*. This implies that the path distance of the leader, $s_{t}^{l}$, must always be greater than or equal to the path distance of the follower, $s_{t}^{f}$. In practical terms, this means that the leader and follower vehicles are determined by searching backwards from the position of the leader. Second, an assumption is made that the radar incorrectly measures the vehicle length when vehicles are distant, a fact which is discussed further in Section 4.1. The imprecise length assessment resulting from distant measurements affects the estimated positions of the vehicle’s front and rear, which subsequently impacts the matching process. To correct for this, the length of vehicles is adjusted to the population mean when it is over 50 m from the radar head. The adjusted length is either subtracted from or added to the radar’s estimation of the vehicle’s front or rear position, depending on whether the vehicle is approaching or moving away from the radar head. The influence of travel direction on the estimation of vehicle front and rear positions is further investigated in Section 4.1.

The geometric configuration of the network is such that a radar hand-off occurs when a vehicle transitions from the coverage area of one radar to another. During this transition, the radar’s reported position of the vehicle, which corresponds to the centroid of the vehicle, may exhibit a bias towards the front or rear of the vehicle. To address this, two steps are taken. First, the covariance matrix *P* is augmented to include more noise in the *s* and *d* dimensions. The additional positional noise is $\sqrt{5/3}$ m in the *s* dimension and 1.5 m in the *d* dimension. Without specific knowledge of the uncertainty, additional position noise is assumed.

Second, this research introduces additional measurements to refine the computation of the $d_{\mathrm{assoc}}^{2}$ distance. The method extends beyond the vehicle centroid C of the vehicle, incorporating the distances to both the front F and rear R of the vehicle. These measurements are distinct in that they modify only the *s* term of the state vector. The measurements are derived using the radars’ reported distance to front and back of the vehicle.

Upon establishing the leader–follower pairs, $d_{\mathrm{assoc}}^{2}$ is calculated between the the three distinct sets of measurements for each pair. The distance is calculated after filtering, making the measurement vector in Equation (1) the filtered $s,\dot{s},d,\dot{d}$. The covariance matrix $S_{i,j}$ in Equation (1) is the posterior covariance matrix *P* from the IMM filter. It is multiplied by the measurement matrix H in order to only include uncertainty in the measurement dimensions, and is augmented with the aforementioned noise in all dimensions.

The ultimate formula for the association distance between a leader and follower is presented as follows:

(13) $$d_{l,f,\mathrm{assoc}}^{2}=\min\left(\left[d_{F-F}^{2},d_{,C-C}^{2},d_{,R-R}^{2}\right]\right)$$

encapsulating the minimum distance from measurements taken at the front, centroid, and back of the vehicles to offer a more encompassing evaluation of the association distance.

In the proposed methodology, Equation (13) is computed for every leader–follower pair at each time step. This computation is performed during the postprocessing stage, which allows for the consideration of some history of a leader–follower pair rather than only a single instance in time. This approach is inspired by [22], where the average distance between trajectories throughout a predefined history is calculated. Specifically, in this work, if $d_{l,f,\mathrm{assoc}}^{2}$ ever falls below the threshold at any point (*p*-value of <0.05), the two trajectories are considered to be the same, thereby emulating real-time operation, making this an “optimistic” association strategy. No attempt to merge tracks is made when the velocity is less that 0.5 m/s, as this can lead to erroneous matches during queuing at traffic signals.

#### 3.5.1. Graph-Based Association Refinement

**Figure 4:**
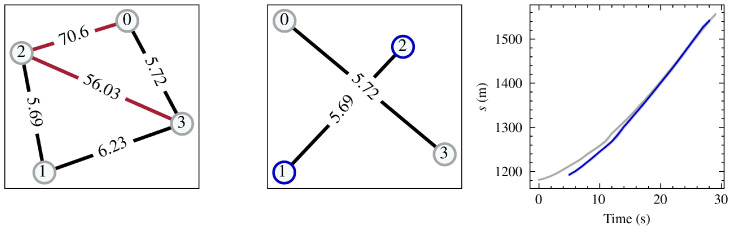
The process of splitting a vehicle graph into optimal subgraphs. The **leftmost** graph represents the result of optimistically joining leader–follower pairs; the red edge represents the additional connection that results from analyzing all co-existing tracklets. The **middle** image displays the result of graph pruning, where the connections between 1 and 3 have been removed along with the connections between 0 and 2 and between 2 and 3, resulting in two separate vehicles. The **rightmost** subplot displays the *s*-position of the vehicles.

The optimistic association described above ultimately forms a set of connections between radar tracklets. To turn the set of connections into individual vehicles, a graph is constructed, G(V,E), where the vertices, *V*, represent radar tracklets and the edges, *E*, are the association distances between the pair of trajectories. To turn G into independent vehicles, all disjoint subgraphs in G are found using a breadth-first search. One such subgraph, $G_{i}$, is shown in the leftmost subplot of Figure 4, where the nodes represent radar tracklets and the edges are the association distances.

In Figure 4, the black edges represent valid edges found using the leader–follower method described earlier. Although this method is computationally efficient, it only considers the history of the nearest tracklet rather than the full history of matched trajectories. This matching approach can lead to suboptimal tracklet matches, particularly when three or more tracklets exist simultaneously for a given trajectory. In such cases, alternative graphs may provide a more optimal solution.

To tackle this problem, the algorithm isolates all subgraphs where three or more tracklets coexist. For each subgraph, it computes the mean of the 2-s moving average of $d_{l,f,\mathrm{assoc}}^{2}$ for all combinations of tracklet pairs in each subgraph, filtering for those that exist in the network at the same time. By taking into account the full history of the matched trajectories rather than just the nearest tracklet, this approach offers a more comprehensive assessment of the association between the tracklets. The red edges in the first subplot in Figure 4 illustrate the additional edges that are added by considering *all* possible combinations of the shown vehicle subgraph. After the additional edges have been added, an iterative algorithm is applied to each subgraph, aiming to remove valid connections until the invalid edges are also eliminated. The middle portion of Figure 4 demonstrates this process, where the connection between tracklets 1 and 3 has been removed, consequently removing the invalid edges between tracklets 0 and 2 and tracklets 2 and 3. The leftmost figure further illustrates the result of this split, showing that although tracklets 1 and 3 have a valid connection due to a relatively short shared history, examining the shared history of tracklets 2 and 0 unveils an invalid connection.

This iterative process of pruning valid connections and reevaluating the subgraph helps to refine the vehicle trajectories and eliminate any erroneous associations introduced by the optimistic matching approach. By meticulously considering the full history of the tracklets and their pairwise associations, the algorithm can identify and remove invalid connections, ultimately yielding more precise and coherent vehicle trajectories.

#### 3.5.2. Tracklet Fusion

Following association, the trajectories are merged using the covariance intersection strategy described in Section 2.2. The 4-s predictions are only kept when no other data are available. Covariance intersection is applied in sequential fashion, as detailed in [28]. After joining the trajectories, they are further smoothed using the Rauch–Tung–Striebel (RTS) smoothing algorithm [38].

Although an IMM filter is used for the initial filtering and prediction, the CALC motion model is utilized to propagate model states during CI and RTS smoothing. The covariance matrix is augmented with additional positional uncertainty prior to the CI stage, which helps the filter to deal with unrealistic jumps in centroid position during radar hand-off caused by the geometric layout of the sensors.

## 4. Dataset Statistics and Evaluation

The proposed method for data association and fusion was applied to historical radar data from two periods in 2023: a large 26-h dataset from March, and a shorter period in October used for validation. The radar data were filtered, associated, and fused in postprocessing, allowing for many of the steps to be massively parallelized, including the IMM, CI, and RTS steps. All methods were altered to run on a GPU, greatly decreasing the processing time for the multi-day dataset from hours to minutes. All code used to process the radar recordings and perform the evaluation is available on Github (https://github.com/UnivOfAlabama-BittleResearchGroup/roadside-radar, accessed on 2 July 2024). The full dataset will be be made available online and linked through the same Github repository.

### 4.1. Positional Accuracy Evaluation

The filter’s accuracy was evaluated using a probe vehicle equipped with a differential GPS (dGPS) with a positional precision of ±0.02 m, as shown in Figure 1. The vehicle traversed the network several times, covering nine segments across four lanes during mid-day traffic, with the average length being 727 m. The dGPS data were mapped to the Frenet coordinate system, akin to the radar, by first converting to UTM coordinates and then using the discretized lanes to map the UTM position to the Frenet frame. After conversion to the Frenet frame, comparable radar traces were identified using the root mean squared error (RMSE) of the position and velocity.

The dGPS and radar data were recorded on separate computing systems, requiring a temporal offset for proper alignment. This alignment was achieved by sweeping a time range and matching each network traversal to the most similar trace in the radar data. The dGPS temporal offset that minimized the overall error across all segments was determined to be −15.5 s relative to the radar.

After establishing the temporal offset, the radar and dGPS data were synchronized by adjusting for time and lane position, resulting in a comprehensive dataset that included all vehicles in the lane during the probe vehicle’s pass as potential matches. The DBSCAN algorithm [39] was applied to filter out vehicle clusters with low RMSE in speed and position, identifying them as “good matches”. These selected radar tracks were then analyzed for positional error, as shown in Table 1.

**Figure 5:**
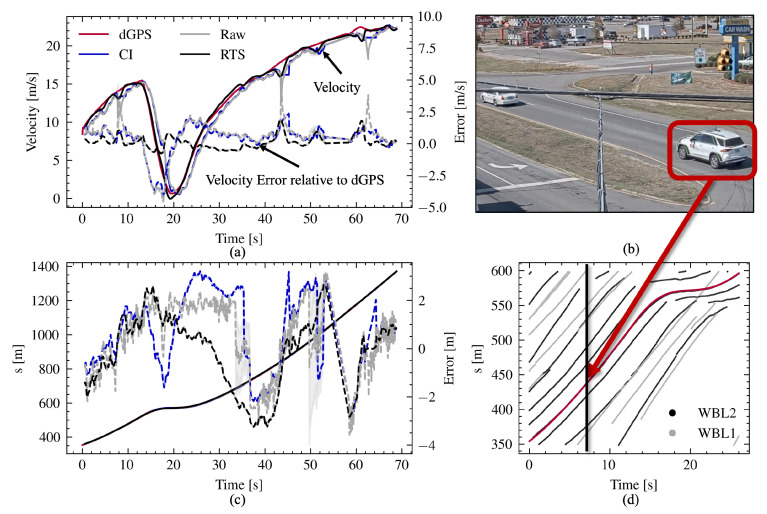
Example of dGPS-equipped probe vehicle matched to radar data in the Frenet frame. The camera image in Subfigure (**b**) displays one vertical slice of the space–time diagram as indicated in Subfigure (**d**), which is displaying both of the WB lanes. The leader vehicle is also visible in both the space–time diagram and the image. Subfigure (**a**) displays the velocity trace of the vehicle as measured by the radar as well as the absolute error resulting from the the CI, Raw, and RTS steps plotted on the secondary y-axis. Subfigure (**c**) shows the centroid position of the vehicle for all radar processing methods and the corresponding error relative to the dGPS data plotted on the secondary y-axis.

Figure 5 provides a visual representation of the matching process, where the radar-detected scene is augmented with roadside camera imagery. The probe vehicle is highlighted in crimson in subplot b, with its Frenet frame position also marked in red on the cropped space-time diagram in subplot c. The traffic in the adjacent lane is shown in light grey. The leader vehicle on WBL2 is apparent in both the image and the space time diagrams. The complete *s* velocity and *s* position time series for the matched sequence are shown in the left-most subplots (a and c), along with to the error, which is shown on the right axis of the position subplot. The shaded regions in light gray represent time periods during which more than one radar was recording the vehicle, meaning that association and fusion occurred. The shown trajectory is the result of merging five radar tracklets from four radars. The artifact of the radar’s internal filtering is clear in the velocity trace, as the measured velocity lags that of the actual vehicle during velocity transients by roughly 1 s. This problem persists in the CI and IMM filtered data, with the CALC RTS filter removing the lag.

Table 1 outlines the error analysis of the tracking system at four key stages: initial radar output (raw); after IMM filtering (IMM); following CI fusion (CI); and after RTS smoothing (RTS). The same steps are shown in Figure 5, with the IMM output removed for brevity. The positional accuracy was measured with reference to the front, rear, and centroid of the probe vehicle. With the dGPS located near the vehicle’s center, the vehicle’s orientation and the measured distances to the front and rear ends were used to pinpoint their positions in both Frenet and UTM coordinates. The evaluation also included an assessment of speed estimation accuracy. Moreover, the average length of the detected tracks was compared to the total length of the road segment, and the count of tracks per segment was tallied for analysis.

Table 1 highlights the increased coverage that fusion provides, with 69% on average of the probe vehicle drives being covered by a single fused track. With no fusion and prediction, a radar’s track only constitutes 29% of the vehicle’s true trajectory. The RTS method yields the minimum RMSE for the centroid position in both coordinate systems as well as for the front position, length, and speed. The CI method provides the minimum RMSE for the back position in both coordinate systems. Overall, the RTS method demonstrates the best performance across most metrics, while the CI method excels in estimating the back position of vehicles. It should be noted that the probe vehicle traces were used to both evaluate the methodology’s accuracy as well as to calibrate the IMM filter measurement uncertainty matrix, which could have led to overestimation of the filtering and fusion accuracy.

**Figure 6:**
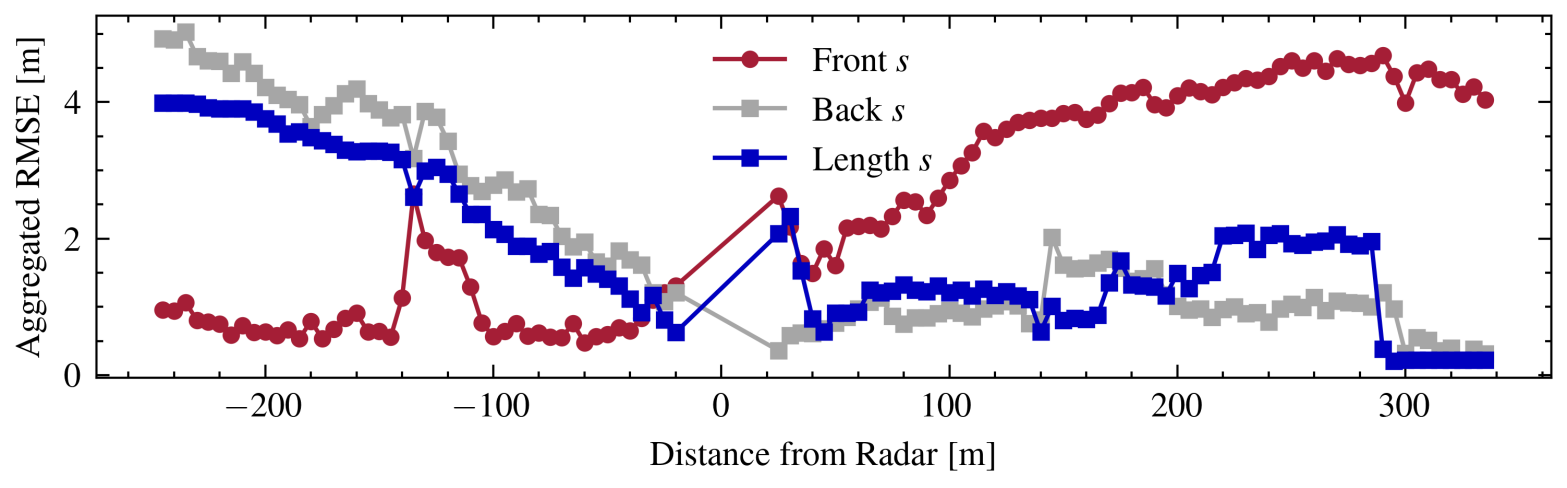
Comparison of the RMSE for the front, back, and length of the vehicle, aggregated by distance from the radar. Negative values indicate a vehicle that is approaching the radar, while positive values indicate a vehicle that is moving away from the radar.

Analysis of the data reveals that the positional accuracy at the rear of the vehicle is consistently higher than at the front in three of the four processing stages. This phenomenon is further investigated in Figure 6, which compiles positional errors relative to the vehicle’s distance from the radar and its direction of travel (either approaching or receding from the radar’s position). Notably, when vehicles are approaching the radar, the RMSE for the front position remains low and stable; in contrast, as vehicles move away from the radar, the error in estimating the front position progressively increases. This trend is attributable to the radar’s reduced lateral visibility of the vehicle as it captures more of the rear profile. The error estimates for the rear position display an opposite pattern, in that they are less accurate when vehicles are approaching the radar and improve as vehicles move away from it. This variation in accuracy is linked to the geometric orientation of the vehicles relative to the radar’s field of view.

### 4.2. Time–Space Glimpse

**Figure 7:**
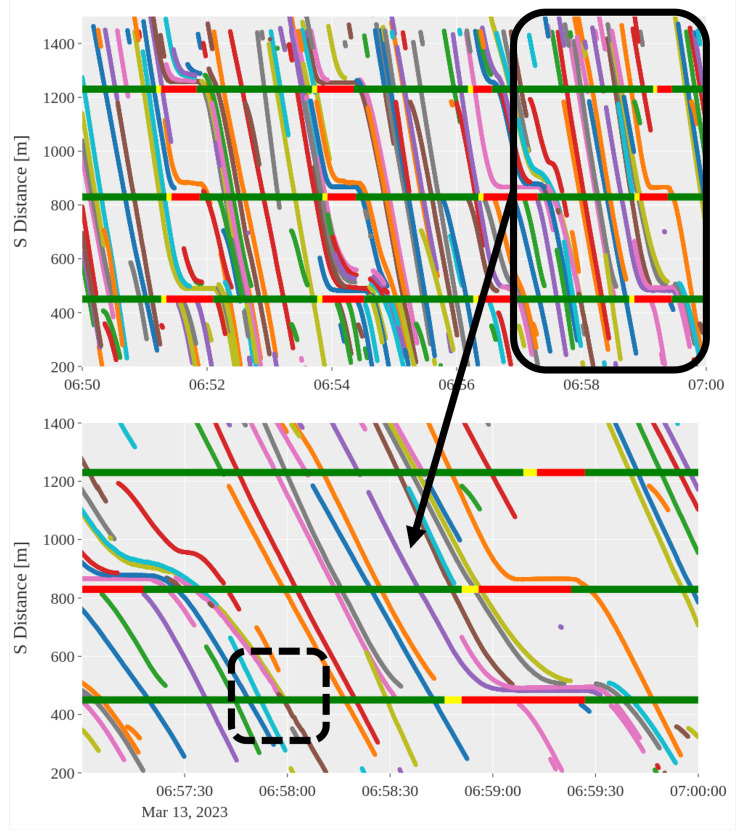
Time–space diagram showing fused trajectories from EB Lane 1 in Figure 2. Plotted underneath are the light states of the three traffic signals in the network at their corresponding *s* position. Trajectories that are the same color represent the same vehicle, with colors being recycled every ten vehicles. The black box in the top figure is exploded in the bottom to show more detail.

A 10-min window of the larger trajectory dataset is depicted in Figure 7, which presents vehicles in EB Lane 1 during the morning of 13 March 2023. A uniform color in the figure signifies that the vehicle trajectory has been synthesized from two or more radar tracks. The states of the three traffic signals, as shown in Figure 2, are superimposed on the figure.

The potential of the merged data is clear, as this 10-min segment has more than 60 vehicles that are in the car-following regime for at least 10 s. The trajectories capture the approach to traffic signals as well as the departure from them. Nonetheless, it is not without issues. The radars often lose track of vehicles when they halt, only re-detecting them when they resume motion, resulting in vehicles being assigned a different track ID. This issue is noticeable in Figure 7 during queuing at red lights, where the four-second trajectory prediction fails to bridge the gap between the trajectories of vehicles coming to a stop at a traffic signal and those departing. The result is overestimation of the total number of vehicles in the network over a certain period. There are also occurrences of unsuccessful association, with one example being failure to associate the brown trajectory with the pink trajectory at time 06:58:00 and S distance 450 m, as shown in the dotted region in the exploded view.

### 4.3. Dataset Description

The dataset spanned over 26 h, from 5 PM on 12 March 2023 to 7:00 PM on 13 March 2023, and contained over 79,000 fused radar tracks resulting from 211,062 radar tracklets. While some vehicles are tracked through the entire corridor, on average each fused vehicle track is composed of 2.6 radar tracklets. Vehicles that have been successfully merged, i.e., from more than one radar track, have an average track length of 672.3 m, compared to 170.8 m for radar tracklets without merging. The median vehicle tracking duration is 18.0 s. Due to the large number of associated tracks, assessing the quality of each one is not feasible. Instead, the mean $d_{assoc}^{2}$ of tracklet associations for each trajectory is used as a proxy for false positives, with 96% of trajectories having a mean $d_{assoc}^{2}$ below the *p*-value cutoff introduced in Section 3.5.

While not comprehensive, Table 2 compares our dataset with two popular trajectory datasets, NGSIM and highD. Our dataset covers a significantly longer time period, including both low traffic volume and rush hour periods. In contrast, highD was recorded only during daytime hours and NGSIM for just a short period. While NGSIM and highD focus on highway driving without side street entries or exits, our dataset captures driving behavior in a network containing three signalized intersections and several unsignalized junctions, as shown in Figure 2. This results in a wide range of captured speeds, with 11.5% of tracked vehicles coming to a complete stop. The values in Table 2 include all tracklets, even if not successfully fused. It is important to note that the tracklet fusion process relies on a probabilistic gating technique applied after IMM filtering, making the values in Table 2 sensitive to the selection of several key parameters. The choice of the *p* value for gating directly impacts the trade-off between the number of vehicles, track length, and the occurrence of erroneous matches; a lower *p* value results in fewer vehicles with longer trajectories but a higher likelihood of incorrect associations. Our chosen *p* value of 0.05 strikes a balance between association confidence and track length, ensuring a robust dataset for analysis.

The comparison of our dataset with NGSIM and highD, shown in Table 2, highlights its unique characteristics and advantages. Thanks to its extended recording duration, our dataset surpasses both NGSIM and highD in terms of temporal coverage, capturing a more comprehensive range of traffic conditions and driving behaviors. Although the number of vehicles in our dataset is lower than in highD, it is significantly higher than in NGSIM. Furthermore, our dataset covers a wide range of recorded distances, providing a diverse set of driving scenarios. The total driven distance and driven time in our dataset are also substantial, enabling in-depth analysis of driver behavior and traffic dynamics. Overall, our dataset offers a unique combination of extended temporal coverage, diverse driving scenarios, and a large number of vehicles, making it a valuable resource for studying real-world driving behavior in signalized corridors.

One of the stated goals of this work was to create a trajectory dataset for the calibration of car-following models. The dataset should encompass a wide range of driving behaviors, including free acceleration, cruising, acceleration, deceleration, following, and standstill [40]. By focusing on such comprehensive trajectories, the calibration process can capture the complex dynamics and interactions between vehicles. To analyze the suitability of this dataset for car-following calibration, a filtering strategy is applied to identify leader–follower pairs that exhibit the desired driving regimes.

First, potential leader–follower pairs are identified based on their time headway, which represents the time gap between the front of the leader vehicle and the front of the follower vehicle. A minimum time headway between 0.5 and 5 s is used to ensure that the follower vehicle is in the following regime. Furthermore, the acceleration and deceleration characteristics of the follower trajectory are examined. The minimum acceleration should be less than −0.2 m/s^2^, indicating that the follower vehicle has experienced deceleration, while the maximum acceleration should be greater than 0.2 m/s^2^, suggesting that the follower vehicle has undergone acceleration. The free acceleration of the follower trajectory, which represents the acceleration when not constrained by a leader, should also be greater than 0.2 m/s^2^, indicating the follower vehicle’s ability to accelerate freely. Lastly, the cruising duration of the follower trajectory should be greater than 2 s, ensuring that the follower vehicle has maintained a relatively constant speed for a sufficient period. After applying these filtering strategies, there are 3,800 trajectory pairs that can be used for calibration, which is substantially more than the 485 trajectories found by Sharma et al. in NGSIM [41].

## 5. Conclusions and Future Work

This paper presents a methodology for extracting vehicle trajectories from six partially overlapping roadside radars in a signalized corridor. The proposed approach combines various techniques, including radar calibration, transformation to the Frenet space, Kalman filtering, short-term prediction, lane classification, association, and a covariance intersection-based approach to track fusion. The effectiveness of the proposed method is validated using a probe vehicle equipped with a differential GPS (dGPS). The results demonstrate that the fused trajectories exhibit improved positional accuracy and coverage compared to the raw radar tracks, with the RTS smoothing method yielding the minimum RMSE for the centroid position and speed.

The extracted and publicized dataset spans 26 h and contains over 79,000 fused radar tracks, with each vehicle track composed of an average of 2.6 radar tracklets. The dataset captures a wide range of driving scenarios, including signalized intersections, merging behavior, and varied speeds, with 11.5% of tracked vehicles coming to a complete stop. Compared to popular trajectory datasets such as NGSIM and highD, this dataset offers extended temporal coverage, a large number of vehicles, and diverse driving conditions. Furthermore, the dataset is suitable for calibrating car-following models, with 3800 filtered leader–follower pairs exhibiting desired driving regimes such as free acceleration, cruising, acceleration, deceleration, following, and standstill, surpassing the 485 trajectories found in the NGSIM dataset. One of the standout features of this dataset is the inclusion of raw radar data and codes along with the processed trajectories, enabling researchers to pursue improved methodologies for prediction, association, and fusion, potentially leveraging techniques from rapidly growing research areas such as deep learning-based trajectory prediction [42].

The proposed framework and the resulting dataset have significant implications for advanced traffic management systems and autonomous vehicle decision-making. By demonstrating the potential of fusing data from multiple roadside radars to extract high-quality vehicle trajectories, this dataset opens up new possibilities for optimizing traffic flow and enhancing road safety. Moreover, the dataset enables a deeper understanding of real-world driving behavior and traffic dynamics in signalized corridors, which is crucial for developing accurate and reliable models for traffic simulation and analysis.

## Figures and Tables

**Table 1:** Average RMSE of the position and velocity at different processing stages of the proposed approach. The minimum RMSE for each position and the maximum coverage are shown in bold.

		RMSE								
	Coverage [%]	Back		Centroid			Front		Length	Speed
Method		(x,y) [m]	s [m]	(x,y) [m]	d [m]	s [m]	(x,y) [m]	s [m]	s [m]	μ [m/s]
Raw	28.87	1.98	1.84	2.10	0.70	1.79	3.34	2.79	1.69	1.01
IMM	31.00	2.04	1.92	2.05	0.67	1.93	3.04	2.95	2.79	0.92
CI	**68.73**	**1.79**	**1.71**	1.91	0.48	1.84	3.06	3.02	**1.33**	1.00
RTS	**68.73**	2.08	2.03	**1.65**	**0.42**	**1.59**	**2.06**	**2.00**	**1.33**	**0.58**

**Table 2:** Comparison of data volume in NGSIM, highD, and our new dataset. Values for NGSIM and highD are from [15].

Attribute	Dataset		
	NGSIM	highD	Current
Recording Duration [h]	1.5	16.5	26.7
Lanes (per direction)	5–6	2–3	2
Recorded Distance [m]	500–640	400–420	34–1182
Vehicles	9206	110,000	79,000 ^1^
Cars	8860	90,000	70,000
Trucks	278	20,000	9500
Driven distance [km]	5071	45,000	32,700
Driven time [h]	174	447	3830

^1^ Merged tracklets.

## Data Availability

All code used to process the radar recordings and perform the evaluation is available on Github (https://github.com/UnivOfAlabama-BittleResearchGroup/roadside-radar, accessed on 2 July 2024). The full dataset will be be made available online and linked through the same Github repository.
